# SAMPI: Protein Identification with Mass Spectra Alignments

**DOI:** 10.1186/1471-2105-8-102

**Published:** 2007-03-26

**Authors:** Hans-Michael Kaltenbach, Andreas Wilke, Sebastian Böcker

**Affiliations:** 1 AG Genominformatik, Technische Fakultät, Universität Bielefeld, PF 100 131, 33501 Bielefeld, Germany; 2 Computation Institute, University of Chicago, Chicago, IL 60637, USA; 3 Lehrstuhl für Bioinformatik, Friedrich-Schiller-Universität Jena, Ernst-Abbe-Platz 2, 07743 Jena, Germany

## Abstract

**Background:**

Mass spectrometry based peptide mass fingerprints (PMFs) offer a fast, efficient, and robust method for protein identification. A protein is digested (usually by trypsin) and its mass spectrum is compared to simulated spectra for protein sequences in a database. However, existing tools for analyzing PMFs often suffer from missing or heuristic analysis of the significance of search results and insufficient handling of missing and additional peaks.

**Results:**

We present an unified framework for analyzing Peptide Mass Fingerprints that offers a number of advantages over existing methods: First, comparison of mass spectra is based on a scoring function that can be custom-designed for certain applications and explicitly takes missing and additional peaks into account. The method is able to simulate almost every additive scoring scheme. Second, we present an efficient deterministic method for assessing the significance of a protein hit, independent of the underlying scoring function and sequence database. We prove the applicability of our approach using biological mass spectrometry data and compare our results to the standard software Mascot.

**Conclusion:**

The proposed framework for analyzing Peptide Mass Fingerprints shows performance comparable to Mascot on small peak lists. Introducing more noise peaks, we are able to keep identification rates at a similar level by using the flexibility introduced by scoring schemes.

## Background

Protein identification using mass spectrometry has become one of the central tools in proteomics and systems biology [1]: With growing protein sequence databases such as SwissProt [2], fast and accurate identification of a sample protein remains a central problem. There are two common strategies for protein identification using mass spectrometry: Peptide Mass Fingerprints [3] and protein identification from peptide sequence information using tandem mass spectrometry [4].

Peptide mass fingerprinting (PMF) is preceded by a protein separation step using gel or chromatographic separation. The separated protein is digested by specific enzymatic cleavage such as tryptic digestion, followed by mass spectrometric measurement of the resulting peptides. The resulting mass spectrum has to be preprocessed into a list of signal peaks that form the input to identification algorithms. In our approach, we concentrate on Matrix Assisted Laser Desorption/Ionization (MALDI) [5], the predominant ionization technique for PMF. This technique produces mainly singly charged ions, allowing us to talk of the mass *m *of a molecule, instead of its mass-to-charge ratio *m*/*z*.

To identify a measured protein from a sequence database, the database sequences are digested in-silico and each predicted peak list is matched and scored with the measured peak list. Usually, computation of the statistical significance should follow, using a statistical background model. Software routinely used for identification of proteins using PMF includes the commercial systems Mascot [6] which uses peak counting together with heuristic information and ProFound [7] which relies on a bayesian scoring scheme. These systems have a comparable performance [8].

In [9], we presented a new approach for PMF protein identification. The approach is based on a re-formulation of the identification problem as a global alignment problem. Further, p-values of identifications are computed using a combinatorial algorithm using uniform character frequencies.

The statistics for p-value computation is extended to a broader class of digestion enzymes and to arbitrary protein sequence models of independently and identically distributed (i.i.d.) amino acids in [10], where this model is also shown to be consistent with corresponding empirical SwissProt data.

Here, we validate the theoretical approach of peak list alignments as introduced in [9] and show the applicability of this approach on real proteomics data. We discuss several aspects of general scoring schemes to be used in peak list alignments; such schemes provide a unified framework that allows emulation and combination of already existing methods and ideas. We demonstrate how missing and additional peaks can explicitly be taken into account and peak intensities can be consistently added into the scoring procedure. We evaluate our method, called *SAMPI *(SAMPI: aligning mass spectra for protein identification), on real proteomics mass spectrometry data and compare the method to PMF identification using the standard software Mascot.

## Results and discussion

To evaluate our method, 375 PMF tryptic mass fingerprints of charge state [*M *+ *H*]^+ ^from an in-house proteomics experiment on the organism *Corynebacterium glutamicum *(Cg) are measured on a Bruker Ultraflex mass spectrometer. The proteins are separated using SDS-PAGE gel electrophoresis before mass measurement. Well-separated spots are digested with trypsin and peptide masses measured by mass spectrometry. For identification, trypsin is set as a cleavage enzyme, Carbamidomethyl is set as a fixed mass modification of ≈ +57 Da for Cysteine, a mass tolerance of 1 Da is set, and no missed cleavages are allowed.

### Processing the raw spectra

**Figure 1:**
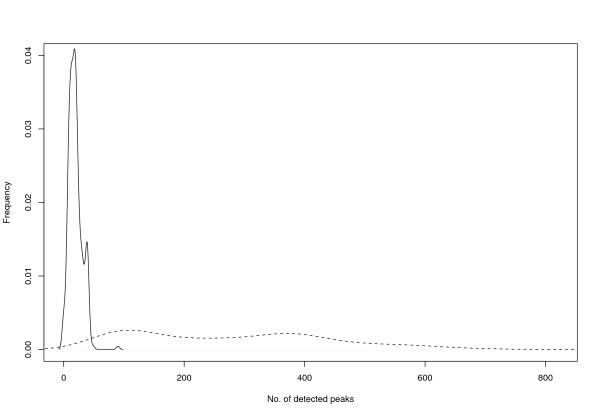
**Peaklist sizes**: Number of detected peaks using the Bruker software (solid) and our peak detection method (dashed).

To assess robustness and flexibility of the method, two different peak lists are derived for each raw spectrum. First, the peak list from the manufacturers peak detection software: It is conservative in picking only the highest abundant peaks; with about 0–90 peaks, 20 on average, these peak lists were comparatively small. Second, we apply a peak detection algorithm developed in our group that derives much larger peak lists of 34–729 peaks, 277 on average. A comparison of the peak list lengths is shown in Figure 1. For unknown reasons, the manufacturers software only delivers 325 peak lists, 9 of which were empty. The other algorithm delivers 375 valid peak lists. For better comparison, we differentiate the peak lists delivered by the algorithm of our group in the following by "PL" and "PL_316_", denoting the whole set of peak lists and the set of peak lists where the manufacturers peak detection also delivers a corresponding nonempty peak list. Due to the different peak detection, the mass ranges for the measured and predicted peak lists were set to 500–3000 Da for the Bruker software, and 800–3000 Da for our peak lists. All peaks outside this range are discarded.

### Databases

Both sets of peak lists are identified using Mascot versions v1.9 and v2.1 and the Gaussian scoring scheme described below with different parameters.

For estimating the false positive rate, we proceed as follows: In a first step, all peak lists are identified using the in-house Cg protein sequence database with 3,510 sequences. The sequence identifiers for each identification are recorded for later comparison.

In a second step, all peak lists are again identified using a concatenation of the SwissProt database, release 48 with 155,824 entries, to the above Cg database. An identification is assumed to be correct if it is the same Cg sequence as recorded before. Conversely, an identification is assumed to be incorrect, if it is not a Cg sequence.

This approach makes it necessary to discard protein sequences that are equal to or highly similar to any Cg sequence from the SwissProt database beforehand. We therefore do an all-against-all comparison of the SwissProt database with 194,317 sequences and the Cg database with BLAST [11]. Sequences from the SwissProt database with an e-value of 10^-30 ^or better are discarded and the remaining 155,824 sequences are appended to the Cg database.

The performance of PMF identification algorithms can thus be evaluated and compared independently on the set of sample mass spectra.

### Results

The runtimes of both SAMPI and Mascot on the modified SwissProt database (FASTA format, no database indexing) and each of the two peak lists including all preprocessing times are listed in Table 1. The results for both versions of Mascot and the Gaussian scheme with several additional/missing scores are listed in Table 2. All parameter combinations were tested with and without use of peak intensities. Not surprising, different parameter sets lead to different numbers of correct identifications. Nevertheless, these numbers do not change rapidly with changing parameters, indicating a robust behavior of the alignment identification procedure. Using the small manufacturer's peak lists, a small penalty of additional and missing peaks yields a comparable number of correct identifications as Mascot. Using peak intensities in the scoring, this number drops considerably. This is most likely due to the fact that these peak lists already consist of the highest abundant peaks, which are now scaled to 1/3 to 1, distorting the relevance of peaks. This problem might be resolved by using a full rank statistic to scale peak intensities as used, e.g., in [12], instead of the implemented robust linear rescaling. Since we describe a proof-of-concept of the peak list alignment framework, we did not investigate this issue further. Using the larger, noisy peak lists results in the complete opposite behavior: Now, without using intensities to discriminate important and non-important peaks, the identification rate drops to about 1/2 for both the Gaussian schemes and Mascot. Additionally using the robust intensities leads to a good identification rate again. Note that now, higher penalties for additional and missing peaks are also helpful.

**Table 1:** Runtimes

	Bruker	PL
SAMPI	23 min (4.2 sec)	99 min (15.8 sec)
Mascot	43.5 min (8 sec)	192 min (30.7 sec)

Runtimes for identification of 325 small (Bruker, Bruk.) and 375 large (PL) peak lists with an average of 20 and 277 peaks, respectively. Values in parentheses are runtimes per spectrum.

**Table 2:** Identification results

		w/out intensity			w/intensity		
		Bruk.	PL	PL_316_	Bruk.	PL	PL_316_
Mascot v1.9		123	58	53	-	-	-
Mascot v2.1		119	59	53	-	-	-

SAMPI							
*c*_1_	*c*_2_						
-0.1	-0.1	112	56	51	72	106	87
-0.2	-0.2	111	56	51	78	96	92
-0.3	-0.3	96	54	48	65	103	98
-0.4	-0.3	89	53	48	52	110	105
-0.4	-0.4	91	54	49	54	108	103
-0.4	-0.5	94	53	48	57	109	104

Number of correctly identified spectra in the Cg+SwissProt database, using Gaussian score, missing/additional peak penalties shown in first two columns. Three different peak lists are considered: Bruk: The peak lists by the Bruker software, PL and PL_316 _which refer to the peak lists by our detection algorithm where PL_316 _are restricted to the 316 out of 325 peak lists that the Bruker software also detected.

**Figure 2:**
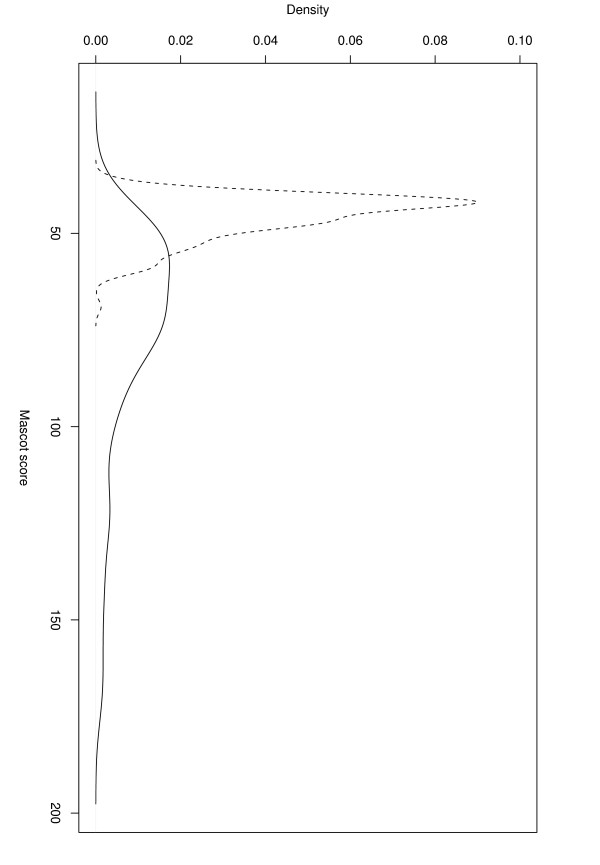
**Score distributions**. *Mascot*: Distribution of Mascot scores of correct (solid line) and incorrect (dashed line) identifications using the Cg+SwissProt database, 1 Da mass tolerance, no missed cleavages, and 316 spectra.

**Figure 3:**
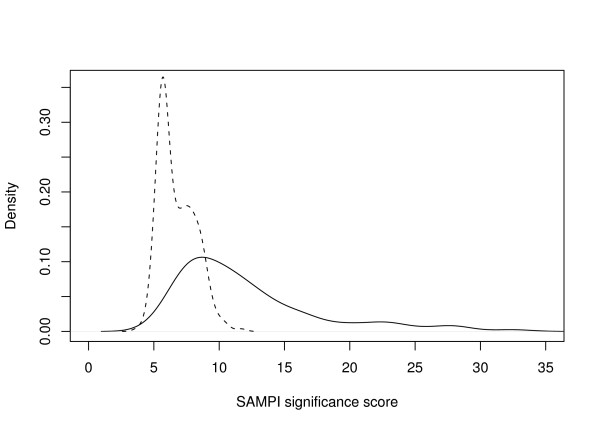
**Score distributions**. *SAMPI*: Distribution of SAMPI scores of correct (solid line) and incorrect (dashed line) identifications using the Cg+SwissProt database, parameter set B, no missed cleavages, and 316 spectra.

We found the score separation of correct and incorrect identifications to be comparable to Mascot (Figures 2 and 3).

## Conclusion

We propose a new formulation of protein identification using Peptide Mass Fingerprinting as an *alignment problem*. We introduce general peak-wise *scoring schemes *and show how these can be used to score two peak lists by dynamic programming. The scoring schemes provide a large amount of flexibility by allowing the user to independently set matching, additional and missing scores. They also allow consistent inclusion of additional features such as peak intensities into the identification process.

A mathematical model based on *random weighted strings *is used to efficiently estimate the statistical significance of an alignment score. This model only needs character frequencies as parameters, which can be estimated even with small sequence databases, allowing the use of species-specific protein data. The *significance score *is then computed to get comparable scores independent of the underlying database and sequence lengths. We also propose a first example of an alignment scoring scheme, called *Gaussian score*, using mass difference and robust intensities. We tested our approach on biological PMF data and compared our results to the standard software Mascot. We were able to correctly identify a comparable number of proteins using peak lists produced by the machine vendor's peak detection software. Using our own peak detection with about 8–10 times as many peaks, we showed the flexibility of our approach by correctly identifying approximately the same number of proteins whereas the performance of Mascot dropped considerably to about half the number of correct identifications.

For the near future, we plan to incorporate missed cleavages into the program. They can easily be handled by the alignment algorithm, but the statistical model has to be extended slightly. Further, we plan to incorporate the method into the ProDB proteomics platform [13] and to compare it to other protein identification tools besides Mascot. As already discussed in the Results and Discussion section, the incorporation of intensity information is not optimal. This is not due to the framework but rather due to the use of scaled intensity values. Nevertheless, using full rank statistics or other probabilistic intensity incorporations are to be investigated in the future. As a last point, the score normalization is not as good as expected, especially on smaller peak lists; the reasons and possible improvements are to be investigated. The flexible alignment framework together with the deterministic, model-based significance computation seems promising, although some improvements are clearly necessary.

## Methods

To identify a measured peak list using peak lists predicted from database sequences, a measure is needed for the similarity of two peak lists. Our scoring of similarity is based on a peak-wise scoring function to score a pair of peaks, one of them possibly being a "gap" peak. The optimal matching of two peak lists can then be computed in a way similar to global sequence alignment.

For computing a statistical significance of an alignment score, we introduce a null-model based on a random protein model and estimate the alignment score distribution.

The general method works as follows, where the individual steps are explained in detail below: In a pre-processing step, a peak list is computed from each entry in the particular protein sequence database; it is called the *predicted peak list *of the sequence. Further, several statistics are computed for later use in the identification's significance estimation. These statistics are the length distribution and the joint length-mass distribution of cleavage fragments. From these, the occurrence probabilities are computed for each possible fragment mass and each protein length contained in the database. Now, the highest scoring protein sequence is computed for each measured spectrum by aligning this spectrum to each predicted spectrum, computing the alignment score and returning the sequence with the highest scoring predicted spectrum. Further, a statistical significance is computed for each such alignment score.

### Spectra alignments

#### Peaks and peak lists

Every peak *p*_*i *_has a mass *m*_*i *_∈ ℳ
 MathType@MTEF@5@5@+=feaafiart1ev1aaatCvAUfKttLearuWrP9MDH5MBPbIqV92AaeXatLxBI9gBamrtHrhAL1wy0L2yHvtyaeHbnfgDOvwBHrxAJfwnaebbnrfifHhDYfgasaacH8akY=wiFfYdH8Gipec8Eeeu0xXdbba9frFj0=OqFfea0dXdd9vqai=hGuQ8kuc9pgc9s8qqaq=dirpe0xb9q8qiLsFr0=vr0=vr0dc8meaabaqaciaacaGaaeqabaWaaeGaeaaakeaaimaacqWFZestaaa@3790@ and possibly other attributes (*a*_*i*,1_, ..., *a*_*i*,*k*_) ∈ A
 MathType@MTEF@5@5@+=feaafiart1ev1aaatCvAUfKttLearuWrP9MDH5MBPbIqV92AaeXatLxBI9gBamrtHrhAL1wy0L2yHvtyaeHbnfgDOvwBHrxAJfwnaebbnrfifHhDYfgasaacH8akY=wiFfYdH8Gipec8Eeeu0xXdbba9frFj0=OqFfea0dXdd9vqai=hGuQ8kuc9pgc9s8qqaq=dirpe0xb9q8qiLsFr0=vr0=vr0dc8meaabaqaciaacaGaaeqabaWaaeGaeaaakeaaimaacqWFaeFqaaa@3821@, *k *≥ 0. A *peak list *S
 MathType@MTEF@5@5@+=feaafiart1ev1aaatCvAUfKttLearuWrP9MDH5MBPbIqV92AaeXatLxBI9gBamrtHrhAL1wy0L2yHvtyaeHbnfgDOvwBHrxAJfwnaebbnrfifHhDYfgasaacH8akY=wiFfYdH8Gipec8Eeeu0xXdbba9frFj0=OqFfea0dXdd9vqai=hGuQ8kuc9pgc9s8qqaq=dirpe0xb9q8qiLsFr0=vr0=vr0dc8meaabaqaciaacaGaaeqabaWaaeGaeaaakeaaimaacqWFse=uaaa@3845@ of length *n *is a list S
 MathType@MTEF@5@5@+=feaafiart1ev1aaatCvAUfKttLearuWrP9MDH5MBPbIqV92AaeXatLxBI9gBamrtHrhAL1wy0L2yHvtyaeHbnfgDOvwBHrxAJfwnaebbnrfifHhDYfgasaacH8akY=wiFfYdH8Gipec8Eeeu0xXdbba9frFj0=OqFfea0dXdd9vqai=hGuQ8kuc9pgc9s8qqaq=dirpe0xb9q8qiLsFr0=vr0=vr0dc8meaabaqaciaacaGaaeqabaWaaeGaeaaakeaaimaacqWFse=uaaa@3845@ = {*p*_1_, ..., *p*_*n*_} of *peaks p*_*i *_∈ ℳ
 MathType@MTEF@5@5@+=feaafiart1ev1aaatCvAUfKttLearuWrP9MDH5MBPbIqV92AaeXatLxBI9gBamrtHrhAL1wy0L2yHvtyaeHbnfgDOvwBHrxAJfwnaebbnrfifHhDYfgasaacH8akY=wiFfYdH8Gipec8Eeeu0xXdbba9frFj0=OqFfea0dXdd9vqai=hGuQ8kuc9pgc9s8qqaq=dirpe0xb9q8qiLsFr0=vr0=vr0dc8meaabaqaciaacaGaaeqabaWaaeGaeaaakeaaimaacqWFZestaaa@3790@ × A
 MathType@MTEF@5@5@+=feaafiart1ev1aaatCvAUfKttLearuWrP9MDH5MBPbIqV92AaeXatLxBI9gBamrtHrhAL1wy0L2yHvtyaeHbnfgDOvwBHrxAJfwnaebbnrfifHhDYfgasaacH8akY=wiFfYdH8Gipec8Eeeu0xXdbba9frFj0=OqFfea0dXdd9vqai=hGuQ8kuc9pgc9s8qqaq=dirpe0xb9q8qiLsFr0=vr0=vr0dc8meaabaqaciaacaGaaeqabaWaaeGaeaaakeaaimaacqWFaeFqaaa@3821@. A peak list is sorted by mass, thus *m*_*i *_<*m*_*j *_if *i *<*j*.

Note that we allow the set A
 MathType@MTEF@5@5@+=feaafiart1ev1aaatCvAUfKttLearuWrP9MDH5MBPbIqV92AaeXatLxBI9gBamrtHrhAL1wy0L2yHvtyaeHbnfgDOvwBHrxAJfwnaebbnrfifHhDYfgasaacH8akY=wiFfYdH8Gipec8Eeeu0xXdbba9frFj0=OqFfea0dXdd9vqai=hGuQ8kuc9pgc9s8qqaq=dirpe0xb9q8qiLsFr0=vr0=vr0dc8meaabaqaciaacaGaaeqabaWaaeGaeaaakeaaimaacqWFaeFqaaa@3821@ of peak attributes to be empty. The simplest type of peak is a peak having only its mass *m *∈ ℝ. A peak with mass and relative intensity could be represented as an element of ℝ × [0,1].

#### Scoring peaks and spectra

Let S
 MathType@MTEF@5@5@+=feaafiart1ev1aaatCvAUfKttLearuWrP9MDH5MBPbIqV92AaeXatLxBI9gBamrtHrhAL1wy0L2yHvtyaeHbnfgDOvwBHrxAJfwnaebbnrfifHhDYfgasaacH8akY=wiFfYdH8Gipec8Eeeu0xXdbba9frFj0=OqFfea0dXdd9vqai=hGuQ8kuc9pgc9s8qqaq=dirpe0xb9q8qiLsFr0=vr0=vr0dc8meaabaqaciaacaGaaeqabaWaaeGaeaaakeaaimaacqWFse=uaaa@3845@_*p *_= {*p*_1_, ..., *p*_*n*_} and S
 MathType@MTEF@5@5@+=feaafiart1ev1aaatCvAUfKttLearuWrP9MDH5MBPbIqV92AaeXatLxBI9gBamrtHrhAL1wy0L2yHvtyaeHbnfgDOvwBHrxAJfwnaebbnrfifHhDYfgasaacH8akY=wiFfYdH8Gipec8Eeeu0xXdbba9frFj0=OqFfea0dXdd9vqai=hGuQ8kuc9pgc9s8qqaq=dirpe0xb9q8qiLsFr0=vr0=vr0dc8meaabaqaciaacaGaaeqabaWaaeGaeaaakeaaimaacqWFse=uaaa@3845@_*m *_= {*p'*_1_, ..., *p'*_*n'*_} be two peak lists of length *n *and *n'*, respectively. For 1 ≤ *i *≤ *n *and 1 ≤ *j *≤ *n'*, let *p*_*i *_∈ ℳ
 MathType@MTEF@5@5@+=feaafiart1ev1aaatCvAUfKttLearuWrP9MDH5MBPbIqV92AaeXatLxBI9gBamrtHrhAL1wy0L2yHvtyaeHbnfgDOvwBHrxAJfwnaebbnrfifHhDYfgasaacH8akY=wiFfYdH8Gipec8Eeeu0xXdbba9frFj0=OqFfea0dXdd9vqai=hGuQ8kuc9pgc9s8qqaq=dirpe0xb9q8qiLsFr0=vr0=vr0dc8meaabaqaciaacaGaaeqabaWaaeGaeaaakeaaimaacqWFZestaaa@3790@ × A
 MathType@MTEF@5@5@+=feaafiart1ev1aaatCvAUfKttLearuWrP9MDH5MBPbIqV92AaeXatLxBI9gBamrtHrhAL1wy0L2yHvtyaeHbnfgDOvwBHrxAJfwnaebbnrfifHhDYfgasaacH8akY=wiFfYdH8Gipec8Eeeu0xXdbba9frFj0=OqFfea0dXdd9vqai=hGuQ8kuc9pgc9s8qqaq=dirpe0xb9q8qiLsFr0=vr0=vr0dc8meaabaqaciaacaGaaeqabaWaaeGaeaaakeaaimaacqWFaeFqaaa@3821@ and *p'*_*j *_∈ ℳ
 MathType@MTEF@5@5@+=feaafiart1ev1aaatCvAUfKttLearuWrP9MDH5MBPbIqV92AaeXatLxBI9gBamrtHrhAL1wy0L2yHvtyaeHbnfgDOvwBHrxAJfwnaebbnrfifHhDYfgasaacH8akY=wiFfYdH8Gipec8Eeeu0xXdbba9frFj0=OqFfea0dXdd9vqai=hGuQ8kuc9pgc9s8qqaq=dirpe0xb9q8qiLsFr0=vr0=vr0dc8meaabaqaciaacaGaaeqabaWaaeGaeaaakeaaimaacqWFZestaaa@3790@ × A
 MathType@MTEF@5@5@+=feaafiart1ev1aaatCvAUfKttLearuWrP9MDH5MBPbIqV92AaeXatLxBI9gBamrtHrhAL1wy0L2yHvtyaeHbnfgDOvwBHrxAJfwnaebbnrfifHhDYfgasaacH8akY=wiFfYdH8Gipec8Eeeu0xXdbba9frFj0=OqFfea0dXdd9vqai=hGuQ8kuc9pgc9s8qqaq=dirpe0xb9q8qiLsFr0=vr0=vr0dc8meaabaqaciaacaGaaeqabaWaaeGaeaaakeaaimaacqWFaeFqaaa@3821@' where we allow the sets of additional peak attributes A
 MathType@MTEF@5@5@+=feaafiart1ev1aaatCvAUfKttLearuWrP9MDH5MBPbIqV92AaeXatLxBI9gBamrtHrhAL1wy0L2yHvtyaeHbnfgDOvwBHrxAJfwnaebbnrfifHhDYfgasaacH8akY=wiFfYdH8Gipec8Eeeu0xXdbba9frFj0=OqFfea0dXdd9vqai=hGuQ8kuc9pgc9s8qqaq=dirpe0xb9q8qiLsFr0=vr0=vr0dc8meaabaqaciaacaGaaeqabaWaaeGaeaaakeaaimaacqWFaeFqaaa@3821@ and A
 MathType@MTEF@5@5@+=feaafiart1ev1aaatCvAUfKttLearuWrP9MDH5MBPbIqV92AaeXatLxBI9gBamrtHrhAL1wy0L2yHvtyaeHbnfgDOvwBHrxAJfwnaebbnrfifHhDYfgasaacH8akY=wiFfYdH8Gipec8Eeeu0xXdbba9frFj0=OqFfea0dXdd9vqai=hGuQ8kuc9pgc9s8qqaq=dirpe0xb9q8qiLsFr0=vr0=vr0dc8meaabaqaciaacaGaaeqabaWaaeGaeaaakeaaimaacqWFaeFqaaa@3821@' to be different. An example would be a measured peak list S
 MathType@MTEF@5@5@+=feaafiart1ev1aaatCvAUfKttLearuWrP9MDH5MBPbIqV92AaeXatLxBI9gBamrtHrhAL1wy0L2yHvtyaeHbnfgDOvwBHrxAJfwnaebbnrfifHhDYfgasaacH8akY=wiFfYdH8Gipec8Eeeu0xXdbba9frFj0=OqFfea0dXdd9vqai=hGuQ8kuc9pgc9s8qqaq=dirpe0xb9q8qiLsFr0=vr0=vr0dc8meaabaqaciaacaGaaeqabaWaaeGaeaaakeaaimaacqWFse=uaaa@3845@_*m*_, with relative intensity and a predicted peak list S
 MathType@MTEF@5@5@+=feaafiart1ev1aaatCvAUfKttLearuWrP9MDH5MBPbIqV92AaeXatLxBI9gBamrtHrhAL1wy0L2yHvtyaeHbnfgDOvwBHrxAJfwnaebbnrfifHhDYfgasaacH8akY=wiFfYdH8Gipec8Eeeu0xXdbba9frFj0=OqFfea0dXdd9vqai=hGuQ8kuc9pgc9s8qqaq=dirpe0xb9q8qiLsFr0=vr0=vr0dc8meaabaqaciaacaGaaeqabaWaaeGaeaaakeaaimaacqWFse=uaaa@3845@_*p *_with the generating string fragment as additional attributes. To compute an optimal matching between peak list S
 MathType@MTEF@5@5@+=feaafiart1ev1aaatCvAUfKttLearuWrP9MDH5MBPbIqV92AaeXatLxBI9gBamrtHrhAL1wy0L2yHvtyaeHbnfgDOvwBHrxAJfwnaebbnrfifHhDYfgasaacH8akY=wiFfYdH8Gipec8Eeeu0xXdbba9frFj0=OqFfea0dXdd9vqai=hGuQ8kuc9pgc9s8qqaq=dirpe0xb9q8qiLsFr0=vr0=vr0dc8meaabaqaciaacaGaaeqabaWaaeGaeaaakeaaimaacqWFse=uaaa@3845@_*p *_and peak list S
 MathType@MTEF@5@5@+=feaafiart1ev1aaatCvAUfKttLearuWrP9MDH5MBPbIqV92AaeXatLxBI9gBamrtHrhAL1wy0L2yHvtyaeHbnfgDOvwBHrxAJfwnaebbnrfifHhDYfgasaacH8akY=wiFfYdH8Gipec8Eeeu0xXdbba9frFj0=OqFfea0dXdd9vqai=hGuQ8kuc9pgc9s8qqaq=dirpe0xb9q8qiLsFr0=vr0=vr0dc8meaabaqaciaacaGaaeqabaWaaeGaeaaakeaaimaacqWFse=uaaa@3845@_*m*_, we first define a scoring function that scores two individual peaks.

A *peak-wise scoring function score *is a function

*score*: (S
 MathType@MTEF@5@5@+=feaafiart1ev1aaatCvAUfKttLearuWrP9MDH5MBPbIqV92AaeXatLxBI9gBamrtHrhAL1wy0L2yHvtyaeHbnfgDOvwBHrxAJfwnaebbnrfifHhDYfgasaacH8akY=wiFfYdH8Gipec8Eeeu0xXdbba9frFj0=OqFfea0dXdd9vqai=hGuQ8kuc9pgc9s8qqaq=dirpe0xb9q8qiLsFr0=vr0=vr0dc8meaabaqaciaacaGaaeqabaWaaeGaeaaakeaaimaacqWFse=uaaa@3845@_*p *_∪ {*ε*}) × (S
 MathType@MTEF@5@5@+=feaafiart1ev1aaatCvAUfKttLearuWrP9MDH5MBPbIqV92AaeXatLxBI9gBamrtHrhAL1wy0L2yHvtyaeHbnfgDOvwBHrxAJfwnaebbnrfifHhDYfgasaacH8akY=wiFfYdH8Gipec8Eeeu0xXdbba9frFj0=OqFfea0dXdd9vqai=hGuQ8kuc9pgc9s8qqaq=dirpe0xb9q8qiLsFr0=vr0=vr0dc8meaabaqaciaacaGaaeqabaWaaeGaeaaakeaaimaacqWFse=uaaa@3845@_*m *_∪ {*ε*}) → ℝ

mapping a predicted and a measured peak to a real value. Here, *ε *denotes a special "gap" peak. For two peaks *p *∈ S
 MathType@MTEF@5@5@+=feaafiart1ev1aaatCvAUfKttLearuWrP9MDH5MBPbIqV92AaeXatLxBI9gBamrtHrhAL1wy0L2yHvtyaeHbnfgDOvwBHrxAJfwnaebbnrfifHhDYfgasaacH8akY=wiFfYdH8Gipec8Eeeu0xXdbba9frFj0=OqFfea0dXdd9vqai=hGuQ8kuc9pgc9s8qqaq=dirpe0xb9q8qiLsFr0=vr0=vr0dc8meaabaqaciaacaGaaeqabaWaaeGaeaaakeaaimaacqWFse=uaaa@3845@_*p *_and *p' *∈ S
 MathType@MTEF@5@5@+=feaafiart1ev1aaatCvAUfKttLearuWrP9MDH5MBPbIqV92AaeXatLxBI9gBamrtHrhAL1wy0L2yHvtyaeHbnfgDOvwBHrxAJfwnaebbnrfifHhDYfgasaacH8akY=wiFfYdH8Gipec8Eeeu0xXdbba9frFj0=OqFfea0dXdd9vqai=hGuQ8kuc9pgc9s8qqaq=dirpe0xb9q8qiLsFr0=vr0=vr0dc8meaabaqaciaacaGaaeqabaWaaeGaeaaakeaaimaacqWFse=uaaa@3845@_*m*_, we say that *score*(*p*, *p'*) is a *matching score*. We call *score*(*p*, *ε*) a *missing score *and *p *a *missing peak*, as it is not matched to any peak in S
 MathType@MTEF@5@5@+=feaafiart1ev1aaatCvAUfKttLearuWrP9MDH5MBPbIqV92AaeXatLxBI9gBamrtHrhAL1wy0L2yHvtyaeHbnfgDOvwBHrxAJfwnaebbnrfifHhDYfgasaacH8akY=wiFfYdH8Gipec8Eeeu0xXdbba9frFj0=OqFfea0dXdd9vqai=hGuQ8kuc9pgc9s8qqaq=dirpe0xb9q8qiLsFr0=vr0=vr0dc8meaabaqaciaacaGaaeqabaWaaeGaeaaakeaaimaacqWFse=uaaa@3845@_*m*_. Similarly, *score*(*ε*, *p'*) is called an *additional score *for an *additional peak p'*. For completeness, we define *score*(*ε*, *ε*) := -∞.

We want to stress that missing and additional peaks are likely to be seen even if the measured spectrum stems from a measurement of a known sequence. Additional peaks, peaks that are seen in the measurement but cannot be explained by the sequence, may simply be chemical noise from the biochemical sample preparation. Missing peaks may occur due to incorrect peak detection or failed ionization of the corresponding fragment. Of course, missing and additional peaks also occur if the measured spectrum does not stem from the sequence under investigation.

#### Example 1 (Peak counting)

*Using only peak mass as attribute, a *peak counting score *could ignore missing and additional peaks, i.e. set score*(*p*, *ε*) = *score*(*ε*, *p'*) = 0, *and give a positive score whenever the difference of the two peak masses m and m' is not too large:*

score(p,p′)={1,if|m−m′|≤δ,−∞,else
 MathType@MTEF@5@5@+=feaafiart1ev1aaatCvAUfKttLearuWrP9MDH5MBPbIqV92AaeXatLxBI9gBaebbnrfifHhDYfgasaacH8akY=wiFfYdH8Gipec8Eeeu0xXdbba9frFj0=OqFfea0dXdd9vqai=hGuQ8kuc9pgc9s8qqaq=dirpe0xb9q8qiLsFr0=vr0=vr0dc8meaabaqaciaacaGaaeqabaqabeGadaaakeaacqWGZbWCcqWGJbWycqWGVbWBcqWGYbGCcqWGLbqzcqGGOaakcqWGWbaCcqGGSaalcuWGWbaCgaqbaiabcMcaPiabg2da9maaceqabaqbaeaabiGaaaqaaiabigdaXiabcYcaSaqaaiabdMgaPjabdAgaMjabcYha8jabd2gaTjabgkHiTiqbd2gaTzaafaGaeiiFaWNaeyizImkcciGae8hTdqMaeiilaWcabaGaeyOeI0IaeyOhIuQaeiilaWcabaGaemyzauMaemiBaWMaem4CamNaemyzaugaaaGaay5Eaaaaaa@5365@

for some positive constant *δ*.

Noting that it would be meaningless to match two pairs of peaks that overcross in mass, we compute the optimal matching between two spectra, i.e. the matching yielding the highest sum of peak-wise scores, as a global alignment, using the well-known dynamic programming recurrence. Let *E*[*i*, *j*] denote the score for the optimal matching between the two spectra up to peaks *p*_*i *_and *p'*_*j*_, respectively. Then the alignment table is computed as

E[0,0]=0,E[i+1,0]=E[i,0]+score(pi+1,ε),E[0,j+1]=E[0,j]+score(ε,p′j+1),E[i+1,j+1]=max⁡{E[i,j+1]+score(pi+1,ε),E[i+1,j]+score(ε,p′j+1),E[i,j]+score(pi+1,p′j+1)}.
 MathType@MTEF@5@5@+=feaafiart1ev1aaatCvAUfKttLearuWrP9MDH5MBPbIqV92AaeXatLxBI9gBaebbnrfifHhDYfgasaacH8akY=wiFfYdH8Gipec8Eeeu0xXdbba9frFj0=OqFfea0dXdd9vqai=hGuQ8kuc9pgc9s8qqaq=dirpe0xb9q8qiLsFr0=vr0=vr0dc8meaabaqaciaacaGaaeqabaqabeGadaaakeaafaqaaeabdaaaaeaacqWGfbqrcqGGBbWwcqaIWaamcqGGSaalcqaIWaamcqGGDbqxaeaacqGH9aqpaeaacqaIWaamcqGGSaalaeaacqWGfbqrcqGGBbWwcqWGPbqAcqGHRaWkcqaIXaqmcqGGSaalcqaIWaamcqGGDbqxaeaacqGH9aqpaeaacqWGfbqrcqGGBbWwcqWGPbqAcqGGSaalcqaIWaamcqGGDbqxcqGHRaWkcqWGZbWCcqWGJbWycqWGVbWBcqWGYbGCcqWGLbqzcqGGOaakcqWGWbaCdaWgaaWcbaGaemyAaKMaey4kaSIaeGymaedabeaakiabcYcaSGGaciab=v7aLjabcMcaPiabcYcaSaqaaiabdweafjabcUfaBjabicdaWiabcYcaSiabdQgaQjabgUcaRiabigdaXiabc2faDbqaaiabg2da9aqaaiabdweafjabcUfaBjabicdaWiabcYcaSiabdQgaQjabc2faDjabgUcaRiabdohaZjabdogaJjabd+gaVjabdkhaYjabdwgaLjabcIcaOiab=v7aLjabcYcaSiqbdchaWzaafaWaaSbaaSqaaiabdQgaQjabgUcaRiabigdaXaqabaGccqGGPaqkcqGGSaalaeaacqWGfbqrcqGGBbWwcqWGPbqAcqGHRaWkcqaIXaqmcqGGSaalcqWGQbGAcqGHRaWkcqaIXaqmcqGGDbqxaeaacqGH9aqpaeaacyGGTbqBcqGGHbqycqGG4baEdaGadeqaauaabaqadeaaaeaacqWGfbqrcqGGBbWwcqWGPbqAcqGGSaalcqWGQbGAcqGHRaWkcqaIXaqmcqGGDbqxcqGHRaWkcqWGZbWCcqWGJbWycqWGVbWBcqWGYbGCcqWGLbqzcqGGOaakcqWGWbaCdaWgaaWcbaGaemyAaKMaey4kaSIaeGymaedabeaakiabcYcaSiab=v7aLjabcMcaPiabcYcaSaqaaiabdweafjabcUfaBjabdMgaPjabgUcaRiabigdaXiabcYcaSiabdQgaQjabc2faDjabgUcaRiabdohaZjabdogaJjabd+gaVjabdkhaYjabdwgaLjabcIcaOiab=v7aLjabcYcaSiqbdchaWzaafaWaaSbaaSqaaiabdQgaQjabgUcaRiabigdaXaqabaGccqGGPaqkcqGGSaalaeaacqWGfbqrcqGGBbWwcqWGPbqAcqGGSaalcqWGQbGAcqGGDbqxcqGHRaWkcqWGZbWCcqWGJbWycqWGVbWBcqWGYbGCcqWGLbqzcqGGOaakcqWGWbaCdaWgaaWcbaGaemyAaKMaey4kaSIaeGymaedabeaakiabcYcaSiqbdchaWzaafaWaaSbaaSqaaiabdQgaQjabgUcaRiabigdaXaqabaGccqGGPaqkaaaacaGL7bGaayzFaaGaeiOla4caaaaa@DD94@

The score *score*(S
 MathType@MTEF@5@5@+=feaafiart1ev1aaatCvAUfKttLearuWrP9MDH5MBPbIqV92AaeXatLxBI9gBamrtHrhAL1wy0L2yHvtyaeHbnfgDOvwBHrxAJfwnaebbnrfifHhDYfgasaacH8akY=wiFfYdH8Gipec8Eeeu0xXdbba9frFj0=OqFfea0dXdd9vqai=hGuQ8kuc9pgc9s8qqaq=dirpe0xb9q8qiLsFr0=vr0=vr0dc8meaabaqaciaacaGaaeqabaWaaeGaeaaakeaaimaacqWFse=uaaa@3845@_*p*_, S
 MathType@MTEF@5@5@+=feaafiart1ev1aaatCvAUfKttLearuWrP9MDH5MBPbIqV92AaeXatLxBI9gBamrtHrhAL1wy0L2yHvtyaeHbnfgDOvwBHrxAJfwnaebbnrfifHhDYfgasaacH8akY=wiFfYdH8Gipec8Eeeu0xXdbba9frFj0=OqFfea0dXdd9vqai=hGuQ8kuc9pgc9s8qqaq=dirpe0xb9q8qiLsFr0=vr0=vr0dc8meaabaqaciaacaGaaeqabaWaaeGaeaaakeaaimaacqWFse=uaaa@3845@_*m*_) of the optimal matching, given in *E*[*n*, *n'*], is called the *alignment score *of the spectra S
 MathType@MTEF@5@5@+=feaafiart1ev1aaatCvAUfKttLearuWrP9MDH5MBPbIqV92AaeXatLxBI9gBamrtHrhAL1wy0L2yHvtyaeHbnfgDOvwBHrxAJfwnaebbnrfifHhDYfgasaacH8akY=wiFfYdH8Gipec8Eeeu0xXdbba9frFj0=OqFfea0dXdd9vqai=hGuQ8kuc9pgc9s8qqaq=dirpe0xb9q8qiLsFr0=vr0=vr0dc8meaabaqaciaacaGaaeqabaWaaeGaeaaakeaaimaacqWFse=uaaa@3845@_*p *_and S
 MathType@MTEF@5@5@+=feaafiart1ev1aaatCvAUfKttLearuWrP9MDH5MBPbIqV92AaeXatLxBI9gBamrtHrhAL1wy0L2yHvtyaeHbnfgDOvwBHrxAJfwnaebbnrfifHhDYfgasaacH8akY=wiFfYdH8Gipec8Eeeu0xXdbba9frFj0=OqFfea0dXdd9vqai=hGuQ8kuc9pgc9s8qqaq=dirpe0xb9q8qiLsFr0=vr0=vr0dc8meaabaqaciaacaGaaeqabaWaaeGaeaaakeaaimaacqWFse=uaaa@3845@_*m*_.

As in the case of sequence alignment, the optimal matching itself can be recovered by backtracking in the dynamic programming table *E*[·, ·]. The alignment score can be computed in time *O*(*n*·*n'*), but faster implementations are possible, using only a diagonal band in *E*[·, ·].

This approach is a standard technique [14,15], and has been successfully applied to such diverse problems as tree ring and liquid chromatography matching [16,17]. A more formal model of peak list alignment can be found in [9].

### Scoring schemes

Although a peak in a measured peak list is described at least by its mass and absolute intensity, most identification algorithms only make use of its mass [18]. This is partly because mass is the most discriminative parameter measured and partly because intensity depends heavily on the actual parameter settings of the machine. The basis for many schemes is the observation that a measurement error between the "real" mass of a molecule and the measured mass can be described by a Gaussian distribution with mean 0 and a standard deviation *sd *dependent on the machine settings and experiment type. The mean might also deviate from 0 if the machine is not calibrated correctly. We will now introduce a family of scoring schemes, the *Gaussian scores*, that will be used in further sections to demonstrate the applicability of our approach. Note however, that the approach is by no means limited to this mass measurement error distribution.

#### Mass difference

The matching score *score*(*p*, *p'*) for two peaks *p *and *p' *with masses *m *and *m'*, respectively, is the probability of a Gaussian distributed random variable *Z *with mean 0 and standard deviation *sd *to exceed ±|*m *- *m'*| in the respective direction, i.e., *score*(*p*, *p'*) = ℙ(|*Z*| ≥ |*m *- *m'*|). This score drops exponentially from 1 to 0 with increasing mass difference. As it is always positive, we set the score to -∞ if it falls below 0.05, that is, if the mass difference exceeds ≈ 2·*sd*. A similar approach is taken in ProFound and the tandem MS software SCOPE [19], whereas Mascot uses a constant positive matching score similar to that of Example 1.

#### Robust incorporation of intensities

In order to incorporate intensities of measured peaks into the scoring, we applied methods from robust statistics successfully used in tandem MS scoring [20]: All peaks in the peak list were ranked according to their absolute intensity. The intensity of the 10% highest abundant peaks were set to 1, the intensity of the 10% lowest abundant peaks to 0. The intensities of the remaining peaks were scaled linearly between 0 and 1. Thus, a very high abundant peak of chemical noise or a small number of wrongly detected, low abundant peaks cannot spoil the interpretation of the whole peak list. Up to this point, we only use intensity values of measured peaks, resulting in an asymmetric scoring scheme. Given an appropriate prediction model [18,21], it would also be possible to incorporate predicted intensities into the scoring. Writing int(*p'*) for a peak's scaled intensity, the matching score is multiplied with (1 + 2 int(*p'*))/3, yielding a factor of 1/3 for lowest and 1 for highest abundant peaks. Again, the approach is suitable for using any other incorporation of intensity information, such as logarithmic transforms as proposed, e.g., in [22].

#### Scoring gap peaks

Using peak-wise scoring schemes allows us to explicitly take additional and missing peaks into account.

For additional peaks, a constant penalty *c*_1 _is given. If intensities are used in the scoring, this penalty is again multiplied by the scaled intensity of the peak: *score*(*ε*, *p'*) = *c*_1_·int(*p'*). Very low abundant peaks are then penalized by 0 and thus simply ignored, and very high abundant peaks that are not explained are highly penalized. For missing peaks, the Gaussian score always gives a constant penalty *c*_2_, but as with matching scores, predicted peak intensities could also be used.

### Background model and significance of alignment scores

To estimate the significance of a score of a measured spectrum and a sequence of certain length *L*, we compute a table of mass occurrence probabilities in random weighted strings in a preprocessing step. Using these probabilities, we get a background model for predicted spectra allowing us to estimate the contribution of each measured peak to the overall alignment score under a well-defined null-model without sampling. We proceed as follows: After introducing a formal model of random protein sequences and their digestion, we compute the joint length-mass distribution of cleavage fragments in such random proteins. We then compute the probability that in a random protein of given length, at least one fragment of certain mass *m *occurs and will thus give rise to a corresponding peak in the predicted spectrum. All these quantities can be computed once in a pre-processing step. Using the mass occurrence probabilities, we estimate the expectation and variance of an alignment score for computing *p*-values of such the score.

#### Weighted strings

A *weighted alphabet *is a finite alphabet Σ together with a *weight *or *mass function μ*: Σ → ∨_>0_, assigning a *mass *to each of its characters. Its domain can be extended to strings *s *∈ Σ* by setting μ(s):=∑i=1|s|μ(si)
 MathType@MTEF@5@5@+=feaafiart1ev1aaatCvAUfKttLearuWrP9MDH5MBPbIqV92AaeXatLxBI9gBaebbnrfifHhDYfgasaacH8akY=wiFfYdH8Gipec8Eeeu0xXdbba9frFj0=OqFfea0dXdd9vqai=hGuQ8kuc9pgc9s8qqaq=dirpe0xb9q8qiLsFr0=vr0=vr0dc8meaabaqaciaacaGaaeqabaqabeGadaaakeaaiiGacqWF8oqBcqGGOaakcqWGZbWCcqGGPaqkcqGG6aGocqGH9aqpdaaeWaqaaiab=X7aTjabcIcaOiabdohaZnaaBaaaleaacqWGPbqAaeqaaOGaeiykaKcaleaacqWGPbqAcqGH9aqpcqaIXaqmaeaacqGG8baFcqWGZbWCcqGG8baFa0GaeyyeIuoaaaa@43B1@. Such strings are called *weighted strings*.

If each character *σ *∈ Σ occurs with probability ℙ(*σ*), we call an i.i.d. sequence of such characters a *random weighted string*. The parameters of this model, i.e., the character frequencies, can be robustly estimated from a sequence database. As they are the only parameters needed for subsequent significance computations, we can use species-specific models where only small sequence databases are available.

Here, we use the alphabet of amino acids of size 20 together with the molecular mass of the amino acids in Dalton (Da), with 1 Da approximately the weight of a neutron. For the computations, we require the masses to be integers. As measured masses are only known to some precision, we can simply scale the real mass by an appropriate precision factor (0.1 or 0.01 for PMF/MALDI) and denote the resulting integer masses by μ*(*σ*). For a precision of 0.1 and character frequencies estimated from SwissProt, release 48, a sample of the weighted amino acid alphabet is given in Table 3:

**Table 3:** Example weighted amino acids

*σ*	A (Ala)	C (Cys)	D (Asp)	E (Glu)	...	Y (Tyr)
*μ*(*σ*)	71.0371	103.0092	115.0269	129.0426	...	163.0633
μ*(*σ*)	710	1030	1150	1290	...	1631
ℙ(*σ*)	0.0785	0.0154	0.0531	0.0661	...	0.0306

Example weighted alphabet for amino acids. Five amino acids with their average molecular weight in Dalton, derived integer molecular weight with precision 0.1 and relative frequencies estimated from SwissProt, release 48.

This model is readily extendible to capture distributions of masses for each character, such that copies of the same amino acid in a protein may have different masses. This allows to model isotopic mass distributions and different amino acid masses due to post-translational modifications such as phosphorylation or methylation.

#### Cleavage schemes

Most proteases cleave a peptide right after the occurrence of a specific *cleavage character *in the amino acid sequence, except in the presence of a *prohibition character *directly following the cleavage character. In the case of trypsin, the set of cleavage characters is Γ = {*K*, *R*} and the set of prohibition characters is Π = {*P*}. Together, Γ and Π form a *cleavage scheme*.

Applying a cleavage scheme on a weighted string results in a *fragmentation *of this string, a set of successive, non-overlapping substrings, the *fragments*.

#### Example 2 (Fragmentation of a string)

*Let *Σ := {*A*, *B*, *C*}, *be a weighted alphabet with weights μ*(*A*) = 1, *μ*(*B*) = 2, *μ*(*C*) = 3, *let *Γ := {*B*}, Π := {*A*} *be a cleavage scheme on *Σ. *Then the string s = ABBACCBACBBB is fragmented into the fragments AB, BACCBACB, B, B of weights μ*(*AB*) = 3, *μ*(*BACCBACB*) = 17 *and μ*(*B*) = 2.

For the sake of brevity, we concentrate on cleavage schemes without prohibition characters. Then, a fragment is simply a string of non-cleavage characters followed by a cleavage character. Generalizations to arbitrary cleavage schemes and more details on the stochastic models and efficient computation can be found in [10].

#### Mass occurrence probabilities

Let *f*^*L*^[*l*, m*] denote the probability that the first fragment of a random weighted string of length *L *has length *l *and integer mass m*. The main recurrence is given for the length-mass distribution of the inner part of a fragment, consisting solely of non-cleavage characters:

f′[l,m∗]=∑σ∉Γf′[l−1,m∗−μ∗(σ)]⋅ℙ(σ),
 MathType@MTEF@5@5@+=feaafiart1ev1aaatCvAUfKttLearuWrP9MDH5MBPbIqV92AaeXatLxBI9gBaebbnrfifHhDYfgasaacH8akY=wiFfYdH8Gipec8Eeeu0xXdbba9frFj0=OqFfea0dXdd9vqai=hGuQ8kuc9pgc9s8qqaq=dirpe0xb9q8qiLsFr0=vr0=vr0dc8meaabaqaciaacaGaaeqabaqabeGadaaakeaacuWGMbGzgaqbaiabcUfaBjabdYgaSjabcYcaSiabd2gaTnaaCaaaleqabaGamaiVgEHiQaaakiabc2faDjabg2da9maaqafabaGafmOzayMbauaacqGGBbWwcqWGSbaBcqGHsislcqaIXaqmcqGGSaalcqWGTbqBdGaGyYbaaSqajaiMbGaGykadaYRHxiIkaaGccqGHsisliiGacqWF8oqBdaahaaWcbeqaaiadaYRHxiIkaaGccqGGOaakcqWFdpWCcqGGPaqkcqGGDbqxcqGHflY1tuuDJXwAK1uy0HMmaeHbfv3ySLgzG0uy0HgiuD3BaGabaiab+LriqjabcIcaOiab=n8aZjabcMcaPaWcbaGae83WdmNaeyycI8Saeu4KdCeabeqdcqGHris5aOGaeiilaWcaaa@6774@

with initial condition *f'*[0, 0] = 1.

The fragment length-mass distribution can be computed by adding the cleavage character to the right and taking care of the finite string length *L*.

#### Lemma 1 (Fragment probabilities)

*The fragment probability f*^*L*^[*l*, m*] *is given for l *<*L by*

fL[l,m∗]=∑σ∉Γf′[l−1,m∗−μ∗(σ)]⋅ℙ(σ)
 MathType@MTEF@5@5@+=feaafiart1ev1aaatCvAUfKttLearuWrP9MDH5MBPbIqV92AaeXatLxBI9gBaebbnrfifHhDYfgasaacH8akY=wiFfYdH8Gipec8Eeeu0xXdbba9frFj0=OqFfea0dXdd9vqai=hGuQ8kuc9pgc9s8qqaq=dirpe0xb9q8qiLsFr0=vr0=vr0dc8meaabaqaciaacaGaaeqabaqabeGadaaakeaacqWGMbGzdaahaaWcbeqaaiabdYeambaakiabcUfaBjabdYgaSjabcYcaSiabd2gaTnaaCaaaleqabaGamaiVgEHiQaaakiabc2faDjabg2da9maaqafabaGafmOzayMbauaacqGGBbWwcqWGSbaBcqGHsislcqaIXaqmcqGGSaalcqWGTbqBdaahaaWcbeqaaiadaYRHxiIkaaGccqGHsisliiGacqWF8oqBdaahaaWcbeqaaiadaYRHxiIkaaGccqGGOaakcqWFdpWCcqGGPaqkcqGGDbqxcqGHflY1tuuDJXwAK1uy0HMmaeHbfv3ySLgzG0uy0HgiuD3BaGabaiab+LriqjabcIcaOiab=n8aZjabcMcaPaWcbaGae83WdmNaeyycI8Saeu4KdCeabeqdcqGHris5aaaa@6476@

*and for the boundary l *= *L by*

fL[L,m∗]=f′[L−1,m∗]+∑σ∈Γf′[L−1,m∗−μ∗(σ)]⋅ℙ(σ).
 MathType@MTEF@5@5@+=feaafiart1ev1aaatCvAUfKttLearuWrP9MDH5MBPbIqV92AaeXatLxBI9gBaebbnrfifHhDYfgasaacH8akY=wiFfYdH8Gipec8Eeeu0xXdbba9frFj0=OqFfea0dXdd9vqai=hGuQ8kuc9pgc9s8qqaq=dirpe0xb9q8qiLsFr0=vr0=vr0dc8meaabaqaciaacaGaaeqabaqabeGadaaakeaacqWGMbGzdaahaaWcbeqaaiabdYeambaakiabcUfaBjabdYeamjabcYcaSiabd2gaTnacaYihaaWcbKaGmgacaYOamaiVgEHiQaaakiabc2faDjabg2da9iqbdAgaMzaafaGaei4waSLaemitaWKaeyOeI0IaeGymaeJaeiilaWIaemyBa02aiaiJCaaaleqcaYyaiaiJcWaG8A4fIOcaaOGaeiyxa0Laey4kaSYaaabuaeaacuWGMbGzgaqbaiabcUfaBjabdYeamjabgkHiTiabigdaXiabcYcaSiabd2gaTnaaCaaaleqabaGamaiVgEHiQaaakiabgkHiTGGaciab=X7aTnaaCaaaleqabaGamaiVgEHiQaaakiabcIcaOiab=n8aZjabcMcaPiabc2faDjabgwSixprr1ngBPrwtHrhAYaqeguuDJXwAKbstHrhAGq1DVbaceaGae4xgHaLaeiikaGIae83WdmNaeiykaKcaleaacqWFdpWCcqGHiiIZcqqHtoWraeqaniabggHiLdGccqGGUaGlaaa@77C8@

The probability that a fragment of length *l *does *not *have mass *m *is computed as f¯
 MathType@MTEF@5@5@+=feaafiart1ev1aaatCvAUfKttLearuWrP9MDH5MBPbIqV92AaeXatLxBI9gBaebbnrfifHhDYfgasaacH8akY=wiFfYdH8Gipec8Eeeu0xXdbba9frFj0=OqFfea0dXdd9vqai=hGuQ8kuc9pgc9s8qqaq=dirpe0xb9q8qiLsFr0=vr0=vr0dc8meaabaqaciaacaGaaeqabaqabeGadaaakeaacuWGMbGzgaqeaaaa@2E19@^*L*^[*l*, m*] = *u*[*l*] - *f*^*L*^[*l*, m*], where *u*[*l*] denotes the probability that a fragment has length *l*; it is a geometric distribution.

Taking the complementary probability f¯
 MathType@MTEF@5@5@+=feaafiart1ev1aaatCvAUfKttLearuWrP9MDH5MBPbIqV92AaeXatLxBI9gBaebbnrfifHhDYfgasaacH8akY=wiFfYdH8Gipec8Eeeu0xXdbba9frFj0=OqFfea0dXdd9vqai=hGuQ8kuc9pgc9s8qqaq=dirpe0xb9q8qiLsFr0=vr0=vr0dc8meaabaqaciaacaGaaeqabaqabeGadaaakeaacuWGMbGzgaqeaaaa@2E19@^*L*^[*l*, m*], we can compute the *mass occurrence probability p*[*L*, m*] that at least one fragment of mass m* occurs in the fragmentation of a random weighted string of length *L*.

#### Lemma 2

*The occurrence probability p*[*L*, m*] = 1 - p¯
 MathType@MTEF@5@5@+=feaafiart1ev1aaatCvAUfKttLearuWrP9MDH5MBPbIqV92AaeXatLxBI9gBaebbnrfifHhDYfgasaacH8akY=wiFfYdH8Gipec8Eeeu0xXdbba9frFj0=OqFfea0dXdd9vqai=hGuQ8kuc9pgc9s8qqaq=dirpe0xb9q8qiLsFr0=vr0=vr0dc8meaabaqaciaacaGaaeqabaqabeGadaaakeaacuWGWbaCgaqeaaaa@2E2D@[*L*, m*] of mass m* *in a random weighted string of length L is given by *p¯
 MathType@MTEF@5@5@+=feaafiart1ev1aaatCvAUfKttLearuWrP9MDH5MBPbIqV92AaeXatLxBI9gBaebbnrfifHhDYfgasaacH8akY=wiFfYdH8Gipec8Eeeu0xXdbba9frFj0=OqFfea0dXdd9vqai=hGuQ8kuc9pgc9s8qqaq=dirpe0xb9q8qiLsFr0=vr0=vr0dc8meaabaqaciaacaGaaeqabaqabeGadaaakeaacuWGWbaCgaqeaaaa@2E2D@[0, m*] = 1 *and*

p¯[L,m∗]=∑l=1Lp¯[L−l,m∗]⋅f¯L[l,m∗].
 MathType@MTEF@5@5@+=feaafiart1ev1aaatCvAUfKttLearuWrP9MDH5MBPbIqV92AaeXatLxBI9gBaebbnrfifHhDYfgasaacH8akY=wiFfYdH8Gipec8Eeeu0xXdbba9frFj0=OqFfea0dXdd9vqai=hGuQ8kuc9pgc9s8qqaq=dirpe0xb9q8qiLsFr0=vr0=vr0dc8meaabaqaciaacaGaaeqabaqabeGadaaakeaacuWGWbaCgaqeaiabcUfaBjabdYeamjabcYcaSiabd2gaTnac0XlhaaWcbKaD8gac0XRamqhVgEHiQaaakiabc2faDjabg2da9maaqahabaGafmiCaaNbaebacqGGBbWwcqWGmbatcqGHsislcqWGSbaBcqGGSaalcqWGTbqBdaahaaWcbeqaaiadaISHxiIkaaGccqGGDbqxcqGHflY1cuWGMbGzgaqeamaaCaaaleqabaGaemitaWeaaOGaei4waSLaemiBaWMaeiilaWIaemyBa02aiaiJCaaaleqcaYyaiaiJcWaGiB4fIOcaaOGaeiyxa0faleaacqWGSbaBcqGH9aqpcqaIXaqmaeaacqWGmbata0GaeyyeIuoakiabc6caUaaa@5FD3@

**Figure 4:**
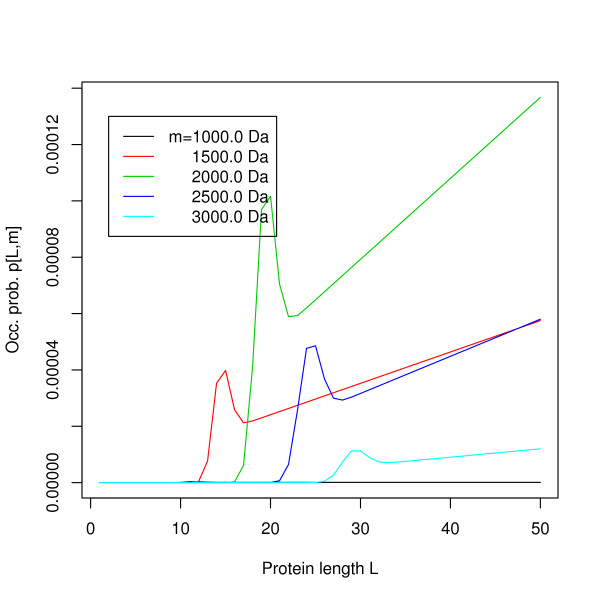
**Occurrence probabilities**. The mass occurrence probabilities *p*[*L*, *m*] for masses. *m *= 1000.0, 1500.0, 2000.0, 2500.0, 3000.0 Da and string length *L *= 1 ... 30. Precision 0.1 Da.

**Figure 5:**
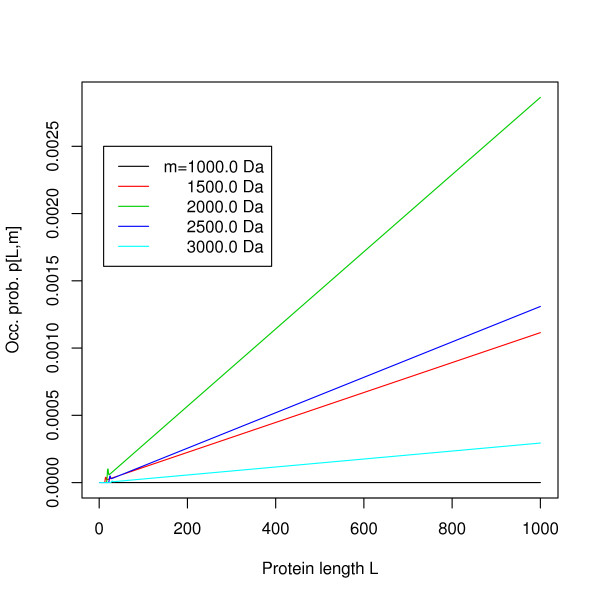
**Occurrence probabilities**. The mass occurrence probabilities *p*[*L*, *m*] for masses. *m *= 1000.0, 1500.0, 2000.0, 2500.0, 3000.0 Da and string length *L *= 1 ... 1000. Precision 0.1 Da.

Both tables have to be computed up to the largest sequence length *L*_*max *_in the sequence database and up to the largest integer fragment mass mmax⁡∗
 MathType@MTEF@5@5@+=feaafiart1ev1aaatCvAUfKttLearuWrP9MDH5MBPbIqV92AaeXatLxBI9gBaebbnrfifHhDYfgasaacH8akY=wiFfYdH8Gipec8Eeeu0xXdbba9frFj0=OqFfea0dXdd9vqai=hGuQ8kuc9pgc9s8qqaq=dirpe0xb9q8qiLsFr0=vr0=vr0dc8meaabaqaciaacaGaaeqabaqabeGadaaakeaacqWGTbqBdaqhaaWcbaGagiyBa0MaeiyyaeMaeiiEaGhabaGamaiSgEHiQaaaaaa@3489@. For PMF using MALDI, *m*_*max *_≈ 3,000 Da and for SwissProt as sequence database, *L*_*max *_≈ 10,000. For a mass precision of one decimal, using doubles, we would need about 30,000·10,000·8 ≈ 2.24 GB of main memory for each table. As f¯
 MathType@MTEF@5@5@+=feaafiart1ev1aaatCvAUfKttLearuWrP9MDH5MBPbIqV92AaeXatLxBI9gBaebbnrfifHhDYfgasaacH8akY=wiFfYdH8Gipec8Eeeu0xXdbba9frFj0=OqFfea0dXdd9vqai=hGuQ8kuc9pgc9s8qqaq=dirpe0xb9q8qiLsFr0=vr0=vr0dc8meaabaqaciaacaGaaeqabaqabeGadaaakeaacuWGMbGzgaqeaaaa@2E19@^*L*^[·, ·] is only needed during computation of p¯
 MathType@MTEF@5@5@+=feaafiart1ev1aaatCvAUfKttLearuWrP9MDH5MBPbIqV92AaeXatLxBI9gBaebbnrfifHhDYfgasaacH8akY=wiFfYdH8Gipec8Eeeu0xXdbba9frFj0=OqFfea0dXdd9vqai=hGuQ8kuc9pgc9s8qqaq=dirpe0xb9q8qiLsFr0=vr0=vr0dc8meaabaqaciaacaGaaeqabaqabeGadaaakeaacuWGWbaCgaqeaaaa@2E2D@[·, ·], only a very small part of about 3–4 MB is required at any time. To efficiently compute the significance of an alignment score, however, the occurrence probability table *p*[·, ·] needs to be kept in memory. Its columns can be computed independently and entries of each column depend smoothly on *L *(the occurrence probability will not change abruptly if the sequence length grows), it is thus sufficient to store only the first 100 entries of each column completely and then store every 25th row, performing a linear interpolation to get intermediate values. Comparing the exact values in each column to the values computed by the described interpolation scheme, we found the interpolation error to be smaller than 10^-9 ^in every case. Note that the interpolation nodes are the exact values, so the interpolation error does not accumulate with growing string length. The mass occurrence probability *p*[*L*, *m*] is given for masses *m *= 1000.0, 1500.0, 2000.0, 2500.0, 3000.0 Da and a precision of 0.1 Da in Figures 4 and 5, for string length up to 50 and 1000, respectively, showing the continuous behavior of the function for *L *> 40. The "hump" at small string lengths can be explained by the fact that for these lengths, the only possible fragment of mass *m *is whole the string itself. For greater string length, the corresponding fragment(s) must be "real" fragments, subject to tighter constraints on their combinatorial character composition, e.g. they must have a cleavage character at the end. This "hump" is located around *L *≈ *m*/*μ*_avg_, where *μ*_avg _denotes the average character mass. For average molecular masses and SwissProt frequencies we have *μ*_avg _≈ 111.2 Da. By further exploiting the fact that *f*^*L*^[*l*, m*] = 0 for *l *> m*/μmin⁡∗
 MathType@MTEF@5@5@+=feaafiart1ev1aaatCvAUfKttLearuWrP9MDH5MBPbIqV92AaeXatLxBI9gBaebbnrfifHhDYfgasaacH8akY=wiFfYdH8Gipec8Eeeu0xXdbba9frFj0=OqFfea0dXdd9vqai=hGuQ8kuc9pgc9s8qqaq=dirpe0xb9q8qiLsFr0=vr0=vr0dc8meaabaqaciaacaGaaeqabaqabeGadaaakeaaiiGacqWF8oqBdaqhaaWcbaGagiyBa0MaeiyAaKMaeiOBa4gabaGamaiSgEHiQaaaaaa@34DF@, where μmin⁡∗
 MathType@MTEF@5@5@+=feaafiart1ev1aaatCvAUfKttLearuWrP9MDH5MBPbIqV92AaeXatLxBI9gBaebbnrfifHhDYfgasaacH8akY=wiFfYdH8Gipec8Eeeu0xXdbba9frFj0=OqFfea0dXdd9vqai=hGuQ8kuc9pgc9s8qqaq=dirpe0xb9q8qiLsFr0=vr0=vr0dc8meaabaqaciaacaGaaeqabaqabeGadaaakeaaiiGacqWF8oqBdaqhaaWcbaGagiyBa0MaeiyAaKMaeiOBa4gabaGamaiSgEHiQaaaaaa@34DF@ is the smallest integer character mass in Σ, both f¯
 MathType@MTEF@5@5@+=feaafiart1ev1aaatCvAUfKttLearuWrP9MDH5MBPbIqV92AaeXatLxBI9gBaebbnrfifHhDYfgasaacH8akY=wiFfYdH8Gipec8Eeeu0xXdbba9frFj0=OqFfea0dXdd9vqai=hGuQ8kuc9pgc9s8qqaq=dirpe0xb9q8qiLsFr0=vr0=vr0dc8meaabaqaciaacaGaaeqabaqabeGadaaakeaacuWGMbGzgaqeaaaa@2E19@^*L*^[*l*, m*] and p¯
 MathType@MTEF@5@5@+=feaafiart1ev1aaatCvAUfKttLearuWrP9MDH5MBPbIqV92AaeXatLxBI9gBaebbnrfifHhDYfgasaacH8akY=wiFfYdH8Gipec8Eeeu0xXdbba9frFj0=OqFfea0dXdd9vqai=hGuQ8kuc9pgc9s8qqaq=dirpe0xb9q8qiLsFr0=vr0=vr0dc8meaabaqaciaacaGaaeqabaqabeGadaaakeaacuWGWbaCgaqeaaaa@2E2D@[*L*, m*] can be computed in time *O*(*L*_max_·mmax⁡∗
 MathType@MTEF@5@5@+=feaafiart1ev1aaatCvAUfKttLearuWrP9MDH5MBPbIqV92AaeXatLxBI9gBaebbnrfifHhDYfgasaacH8akY=wiFfYdH8Gipec8Eeeu0xXdbba9frFj0=OqFfea0dXdd9vqai=hGuQ8kuc9pgc9s8qqaq=dirpe0xb9q8qiLsFr0=vr0=vr0dc8meaabaqaciaacaGaaeqabaqabeGadaaakeaacqWGTbqBdaqhaaWcbaGagiyBa0MaeiyyaeMaeiiEaGhabaGamaiSgEHiQaaaaaa@3489@). We would like to refer the interested reader to [10] for details and proofs on the memory- and time efficient implementation.

#### Alignment score distribution

The alignment score distribution is efficiently and deterministically estimated by adding the contribution of each peak to the overall alignment score. To compute the contribution of each measured peak *p'*_*j*_, ∈ S
 MathType@MTEF@5@5@+=feaafiart1ev1aaatCvAUfKttLearuWrP9MDH5MBPbIqV92AaeXatLxBI9gBamrtHrhAL1wy0L2yHvtyaeHbnfgDOvwBHrxAJfwnaebbnrfifHhDYfgasaacH8akY=wiFfYdH8Gipec8Eeeu0xXdbba9frFj0=OqFfea0dXdd9vqai=hGuQ8kuc9pgc9s8qqaq=dirpe0xb9q8qiLsFr0=vr0=vr0dc8meaabaqaciaacaGaaeqabaWaaeGaeaaakeaaimaacqWFse=uaaa@3845@_*m*_, let Uj
 MathType@MTEF@5@5@+=feaafiart1ev1aaatCvAUfKttLearuWrP9MDH5MBPbIqV92AaeXatLxBI9gBamrtHrhAL1wy0L2yHvtyaeHbnfgDOvwBHrxAJfwnaebbnrfifHhDYfgasaacH8akY=wiFfYdH8Gipec8Eeeu0xXdbba9frFj0=OqFfea0dXdd9vqai=hGuQ8kuc9pgc9s8qqaq=dirpe0xb9q8qiLsFr0=vr0=vr0dc8meaabaqaciaacaGaaeqabaWaaeGaeaaakeaaimqacqWFueFvdaWgaaWcbaGaemOAaOgabeaaaaa@39D3@_*j *_denote the *support *of peak *p'*_*j*_, that is the set of integer masses m* for which a predicted peak *p *having integer mass m* would contribute a positive matching score *score*(*p*, *p'*_*j*_) > 0. Let Xjmatch(L)
 MathType@MTEF@5@5@+=feaafiart1ev1aaatCvAUfKttLearuWrP9MDH5MBPbIqV92AaeXatLxBI9gBaebbnrfifHhDYfgasaacH8akY=wiFfYdH8Gipec8Eeeu0xXdbba9frFj0=OqFfea0dXdd9vqai=hGuQ8kuc9pgc9s8qqaq=dirpe0xb9q8qiLsFr0=vr0=vr0dc8meaabaqaciaacaGaaeqabaqabeGadaaakeaacqWGybawdaqhaaWcbaGaemOAaOgabaacbaGae8xBa0Mae8xyaeMae8hDaqNae83yamMae8hAaGgaaOGaeiikaGIaemitaWKaeiykaKcaaa@3908@ be the random variable that contains the sum of the matching scores over all peaks *p *in any spectrum S
 MathType@MTEF@5@5@+=feaafiart1ev1aaatCvAUfKttLearuWrP9MDH5MBPbIqV92AaeXatLxBI9gBamrtHrhAL1wy0L2yHvtyaeHbnfgDOvwBHrxAJfwnaebbnrfifHhDYfgasaacH8akY=wiFfYdH8Gipec8Eeeu0xXdbba9frFj0=OqFfea0dXdd9vqai=hGuQ8kuc9pgc9s8qqaq=dirpe0xb9q8qiLsFr0=vr0=vr0dc8meaabaqaciaacaGaaeqabaWaaeGaeaaakeaaimaacqWFse=uaaa@3845@_*p *_generated by a random weighted string of length *L *that have masses in U′j
 MathType@MTEF@5@5@+=feaafiart1ev1aaatCvAUfKttLearuWrP9MDH5MBPbIqV92AaeXatLxBI9gBamrtHrhAL1wy0L2yHvtyaeHbnfgDOvwBHrxAJfwnaebbnrfifHhDYfgasaacH8akY=wiFfYdH8Gipec8Eeeu0xXdbba9frFj0=OqFfea0dXdd9vqai=hGuQ8kuc9pgc9s8qqaq=dirpe0xb9q8qiLsFr0=vr0=vr0dc8meaabaqaciaacaGaaeqabaWaaeGaeaaakeaaimqacuWFueFvgaqbamaaBaaaleaacqWGQbGAaeqaaaaa@39DF@. The expectation and variance of this random variable are then given by

E(Xjmatch(L))=∑m∗∈U′jp[L,m∗]⋅score(p,p′j)
 MathType@MTEF@5@5@+=feaafiart1ev1aaatCvAUfKttLearuWrP9MDH5MBPbIqV92AaeXatLxBI9gBaebbnrfifHhDYfgasaacH8akY=wiFfYdH8Gipec8Eeeu0xXdbba9frFj0=OqFfea0dXdd9vqai=hGuQ8kuc9pgc9s8qqaq=dirpe0xb9q8qiLsFr0=vr0=vr0dc8meaabaqaciaacaGaaeqabaqabeGadaaakeaatuuDJXwAK1uy0HMmaeHbfv3ySLgzG0uy0HgiuD3BaGabaiab=ri8fHqaaiab+HcaOGqaciab9HfaynaaDaaaleaacqWGQbGAaeaacqGFTbqBcqGFHbqycqGF0baDcqGFJbWycqGFObaAaaGccqGGOaakcqWGmbatcqGGPaqkcqGGPaqkcqGH9aqpdaaeqbqaaiabdchaWjabcUfaBjabdYeamjabcYcaSiabd2gaTnaaCaaaleqabaGamaiVgEHiQaaakiabc2faDjabgwSixlabdohaZjabdogaJjabd+gaVjabdkhaYjabdwgaLjabcIcaOiabdchaWjabcYcaSiqbdchaWzaafaWaaSbaaSqaaiabdQgaQbqabaGccqGGPaqkaSqaaiabd2gaTnacaclhaaadbKaGWgacacRamaiVgEHiQaaaliabgIGioprtHrhAL1wy0L2yHvtyaeXbnfgDOvwBHrxAJfwnaGqbbiqb8rr8vzaafaWaaSbaaWqaaiabdQgaQbqabaaaleqaniabggHiLdaaaa@79E0@

Var(Xjmatch(L))=∑m∗∈U′jp[L,m∗]⋅score(p,p′j)2−(E(Xjmatch(L)))2
 MathType@MTEF@5@5@+=feaafiart1ev1aaatCvAUfKttLearuWrP9MDH5MBPbIqV92AaeXatLxBI9gBaebbnrfifHhDYfgasaacH8akY=wiFfYdH8Gipec8Eeeu0xXdbba9frFj0=OqFfea0dXdd9vqai=hGuQ8kuc9pgc9s8qqaq=dirpe0xb9q8qiLsFr0=vr0=vr0dc8meaabaqaciaacaGaaeqabaqabeGadaaakeaaieaacqWFwbGvcqWFHbqycqWFYbGCcqWFOaakieGacqGFybawdaqhaaWcbaGaemOAaOgabaGae8xBa0Mae8xyaeMae8hDaqNae83yamMae8hAaGgaaOGaeiikaGIaemitaWKaeiykaKIaeiykaKIaeyypa0ZaaabuaeaacqWGWbaCcqGGBbWwcqWGmbatcqGGSaalcqWGTbqBdaahaaWcbeqaaiadaYRHxiIkaaGccqGGDbqxcqGHflY1cqWGZbWCcqWGJbWycqWGVbWBcqWGYbGCcqWGLbqzcqGGOaakcqWGWbaCcqGGSaalcuWGWbaCgaqbamaaBaaaleaacqWGQbGAaeqaaOGaeiykaKYaaWbaaSqabeaacqaIYaGmaaaabaGaemyBa02aiaiVCaaameqcaYBaiaiVcWaG8A4fIOcaaSGaeyicI48enfgDOvwBHrxAJfwnHbqeg0uy0HwzTfgDPnwy1aaceeGaf0hfXxLbauaadaWgaaadbaGaemOAaOgabeaaaSqab0GaeyyeIuoakiabgkHiTmaabmaabaWefv3ySLgznfgDOjdarCqr1ngBPrginfgDObcv39gaiuaacqaFecFrcqWFOaakcqGFybawdaqhaaWcbaGaemOAaOgabaGae8xBa0Mae8xyaeMae8hDaqNae83yamMae8hAaGgaaOGaeiikaGIaemitaWKaeiykaKIaeiykaKcacaGLOaGaayzkaaWaaWbaaSqabeaacqaIYaGmaaaaaa@909A@

Similarly, we define random variables Xjadd(L)
 MathType@MTEF@5@5@+=feaafiart1ev1aaatCvAUfKttLearuWrP9MDH5MBPbIqV92AaeXatLxBI9gBaebbnrfifHhDYfgasaacH8akY=wiFfYdH8Gipec8Eeeu0xXdbba9frFj0=OqFfea0dXdd9vqai=hGuQ8kuc9pgc9s8qqaq=dirpe0xb9q8qiLsFr0=vr0=vr0dc8meaabaqaciaacaGaaeqabaqabeGadaaakeaacqWGybawdaqhaaWcbaGaemOAaOgabaacbaGae8xyaeMae8hzaqMae8hzaqgaaOGaeiikaGIaemitaWKaeiykaKcaaa@3636@ for the additional scores. Assuming independence of peaks, the overall matching and additional scores are simply the sum of these scores:

Xmatch(L)=∑j=1n′Xjmatch(L)andXadd(L)=∑j=1n′Xjadd(L)
 MathType@MTEF@5@5@+=feaafiart1ev1aaatCvAUfKttLearuWrP9MDH5MBPbIqV92AaeXatLxBI9gBaebbnrfifHhDYfgasaacH8akY=wiFfYdH8Gipec8Eeeu0xXdbba9frFj0=OqFfea0dXdd9vqai=hGuQ8kuc9pgc9s8qqaq=dirpe0xb9q8qiLsFr0=vr0=vr0dc8meaabaqaciaacaGaaeqabaqabeGadaaakeaafaqabeqadaaabaGaemiwaG1aaWbaaSqabeaaieaacqWFTbqBcqWFHbqycqWF0baDcqWFJbWycqWFObaAaaGccqGGOaakcqWGmbatcqGGPaqkcqGH9aqpdaaeWbqaaiabdIfaynaaDaaaleaacqWGQbGAaeaacqWFTbqBcqWFHbqycqWF0baDcqWFJbWycqWFObaAaaaabaGaemOAaOMaeyypa0JaeGymaedabaGafmOBa4Mbauaaa0GaeyyeIuoakiabcIcaOiabdYeamjabcMcaPaqaaiab=fgaHjab=5gaUjab=rgaKbqaaiabdIfaynaaCaaaleqabaGae8xyaeMae8hzaqMae8hzaqgaaOGaeiikaGIaemitaWKaeiykaKIaeyypa0ZaaabCaeaacqWGybawdaqhaaWcbaGaemOAaOgabaGae8xyaeMae8hzaqMae8hzaqgaaaqaaiabdQgaQjabg2da9iabigdaXaqaaiqbd6gaUzaafaaaniabggHiLdGccqGGOaakcqWGmbatcqGGPaqkaaaaaa@69A9@

The missing scores are given for all masses that are not inside the support of any measured peak:

Xmiss=∑m∗∉∪U′jXm∗miss
 MathType@MTEF@5@5@+=feaafiart1ev1aaatCvAUfKttLearuWrP9MDH5MBPbIqV92AaeXatLxBI9gBaebbnrfifHhDYfgasaacH8akY=wiFfYdH8Gipec8Eeeu0xXdbba9frFj0=OqFfea0dXdd9vqai=hGuQ8kuc9pgc9s8qqaq=dirpe0xb9q8qiLsFr0=vr0=vr0dc8meaabaqaciaacaGaaeqabaqabeGadaaakeaacqWGybawdaahaaWcbeqaaGqaaiab=1gaTjab=LgaPjab=nhaZjab=nhaZbaakiabg2da9maaqafabaGaemiwaG1aa0baaSqaaiabd2gaTnaaCaaameqabaGamaiPgEHiQaaaaSqaaiab=1gaTjab=LgaPjab=nhaZjab=nhaZbaaaeaacqWGTbqBdGaGmYbaaWqajaiJbGaGmkadaYRHxiIkaaWccqGHjiYZdaWeaaqaamrtHrhAL1wy0L2yHvtyaeHbnfgDOvwBHrxAJfwnaGabbiqb+rr8vzaafaWaaSbaaWqaaiabdQgaQbqabaaabeqab4GaeSOkIufaaSqab0GaeyyeIuoaaaa@58A1@

We omit the details for additional and missing peaks and again refer the interested reader to [9]. The alignment score *score*(S
 MathType@MTEF@5@5@+=feaafiart1ev1aaatCvAUfKttLearuWrP9MDH5MBPbIqV92AaeXatLxBI9gBamrtHrhAL1wy0L2yHvtyaeHbnfgDOvwBHrxAJfwnaebbnrfifHhDYfgasaacH8akY=wiFfYdH8Gipec8Eeeu0xXdbba9frFj0=OqFfea0dXdd9vqai=hGuQ8kuc9pgc9s8qqaq=dirpe0xb9q8qiLsFr0=vr0=vr0dc8meaabaqaciaacaGaaeqabaWaaeGaeaaakeaaimaacqWFse=uaaa@3845@_*p*_, S
 MathType@MTEF@5@5@+=feaafiart1ev1aaatCvAUfKttLearuWrP9MDH5MBPbIqV92AaeXatLxBI9gBamrtHrhAL1wy0L2yHvtyaeHbnfgDOvwBHrxAJfwnaebbnrfifHhDYfgasaacH8akY=wiFfYdH8Gipec8Eeeu0xXdbba9frFj0=OqFfea0dXdd9vqai=hGuQ8kuc9pgc9s8qqaq=dirpe0xb9q8qiLsFr0=vr0=vr0dc8meaabaqaciaacaGaaeqabaWaaeGaeaaakeaaimaacqWFse=uaaa@3845@_*m*_) for a measured spectrum S
 MathType@MTEF@5@5@+=feaafiart1ev1aaatCvAUfKttLearuWrP9MDH5MBPbIqV92AaeXatLxBI9gBamrtHrhAL1wy0L2yHvtyaeHbnfgDOvwBHrxAJfwnaebbnrfifHhDYfgasaacH8akY=wiFfYdH8Gipec8Eeeu0xXdbba9frFj0=OqFfea0dXdd9vqai=hGuQ8kuc9pgc9s8qqaq=dirpe0xb9q8qiLsFr0=vr0=vr0dc8meaabaqaciaacaGaaeqabaWaaeGaeaaakeaaimaacqWFse=uaaa@3845@_*m *_and a random predicted spectrum S
 MathType@MTEF@5@5@+=feaafiart1ev1aaatCvAUfKttLearuWrP9MDH5MBPbIqV92AaeXatLxBI9gBamrtHrhAL1wy0L2yHvtyaeHbnfgDOvwBHrxAJfwnaebbnrfifHhDYfgasaacH8akY=wiFfYdH8Gipec8Eeeu0xXdbba9frFj0=OqFfea0dXdd9vqai=hGuQ8kuc9pgc9s8qqaq=dirpe0xb9q8qiLsFr0=vr0=vr0dc8meaabaqaciaacaGaaeqabaWaaeGaeaaakeaaimaacqWFse=uaaa@3845@_*p *_generated by a random weighted string of length *L *is finally given by

*score*(S
 MathType@MTEF@5@5@+=feaafiart1ev1aaatCvAUfKttLearuWrP9MDH5MBPbIqV92AaeXatLxBI9gBamrtHrhAL1wy0L2yHvtyaeHbnfgDOvwBHrxAJfwnaebbnrfifHhDYfgasaacH8akY=wiFfYdH8Gipec8Eeeu0xXdbba9frFj0=OqFfea0dXdd9vqai=hGuQ8kuc9pgc9s8qqaq=dirpe0xb9q8qiLsFr0=vr0=vr0dc8meaabaqaciaacaGaaeqabaWaaeGaeaaakeaaimaacqWFse=uaaa@3845@_*p*_, S
 MathType@MTEF@5@5@+=feaafiart1ev1aaatCvAUfKttLearuWrP9MDH5MBPbIqV92AaeXatLxBI9gBamrtHrhAL1wy0L2yHvtyaeHbnfgDOvwBHrxAJfwnaebbnrfifHhDYfgasaacH8akY=wiFfYdH8Gipec8Eeeu0xXdbba9frFj0=OqFfea0dXdd9vqai=hGuQ8kuc9pgc9s8qqaq=dirpe0xb9q8qiLsFr0=vr0=vr0dc8meaabaqaciaacaGaaeqabaWaaeGaeaaakeaaimaacqWFse=uaaa@3845@_*m*_) = *X*^match^(*L*) + *X*^add^(*L*) + *X*^miss^.

As *score*(S
 MathType@MTEF@5@5@+=feaafiart1ev1aaatCvAUfKttLearuWrP9MDH5MBPbIqV92AaeXatLxBI9gBamrtHrhAL1wy0L2yHvtyaeHbnfgDOvwBHrxAJfwnaebbnrfifHhDYfgasaacH8akY=wiFfYdH8Gipec8Eeeu0xXdbba9frFj0=OqFfea0dXdd9vqai=hGuQ8kuc9pgc9s8qqaq=dirpe0xb9q8qiLsFr0=vr0=vr0dc8meaabaqaciaacaGaaeqabaWaaeGaeaaakeaaimaacqWFse=uaaa@3845@_*p*_, S
 MathType@MTEF@5@5@+=feaafiart1ev1aaatCvAUfKttLearuWrP9MDH5MBPbIqV92AaeXatLxBI9gBamrtHrhAL1wy0L2yHvtyaeHbnfgDOvwBHrxAJfwnaebbnrfifHhDYfgasaacH8akY=wiFfYdH8Gipec8Eeeu0xXdbba9frFj0=OqFfea0dXdd9vqai=hGuQ8kuc9pgc9s8qqaq=dirpe0xb9q8qiLsFr0=vr0=vr0dc8meaabaqaciaacaGaaeqabaWaaeGaeaaakeaaimaacqWFse=uaaa@3845@_*m*_) is the sum of nearly independent random variables, we can expect it to have a normal distribution for reasonable scoring schemes and peak lists. This distribution is completely determined by its expectation and variance.

Note that the alignment algorithm computes an optimal one-to-one peak matching score, whereas the estimation procedure corresponds to a many-to-one matching of peaks. As shown below, neither the peak independence assumptions nor the one-to-one peak matching are a problem in practice, as violations of either assumption do not contribute enough to alter the score distribution noticably.

**Figure 6:**
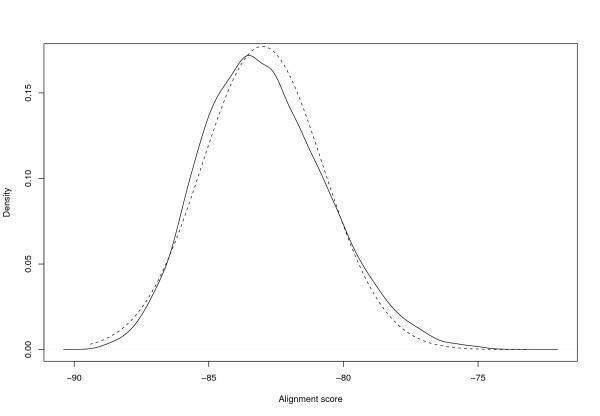
**Alignment score distribution**. **A**: Solid line: Densities of empirical alignment score distribution using 10,000 randomly generated protein sequences of length 250 with SwissProt amino acid frequencies. Dashed line: Density of approximating normal distribution with parameters computed as described in the text. Both alignments for one measured spectrum and SAMPI score with parameter set A.

**Figure 7:**
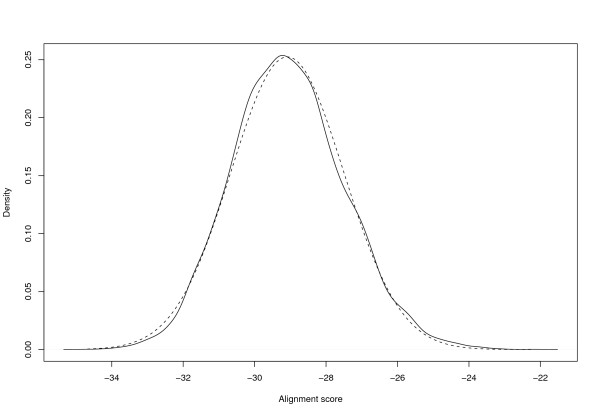
**Alignment score distribution**. **B**: Solid line: Densities of empirical alignment score distribution using 10,000 randomly generated protein sequences of length 250 with SwissProt amino acid frequencies. Dashed line: Density of approximating normal distribution with parameters computed as described in the text. Both alignments for one measured spectrum and SAMPI score with parameter set B.

We tested the two assumptions using two different parameter sets *A *and *B *for the Gaussian score given in Table 4, and computing the alignment scores of 10,000 random amino acid sequences of length 250 and a randomly chosen measured spectrum from our dataset. We found the estimated distributions in good agreement with their empirical counterparts, as shown in Figures 6 and 7.

**Table 4:** Parameter sets for evaluation

Parameter	std. dev. *sd*	missing score	additional score	intensity used
A	0.8	-0.1	-0.1	No
B	0.8	-0.4	-0.3	Yes

The two parameter sets A and B used for the evaluation. Shown are the standard deviation for the Gaussian mass deviance distribution, the missing and additional scores and whether relative peak intensities are used.

#### Using significance as score

As the alignment score is an additive score, its value and distribution is dependent on the number of peaks in the measured and predicted spectra. This makes it difficult to compare alignment scores for different measured spectra and sequence lengths.

To avoid these problems, we will not use the alignment score itself, but its significance to rank the candidate sequences, a method previously shown to be effective for tandem MS data [20]. For each pair of measured and predicted spectra, the alignment score is computed, its distribution is estimated using the method described, and the p-value – the probability that a random sequence of the same length as the aligned sequence gives an alignment score at least as good as the computed one – is computed from this distribution. We then take – log_10_(p-val.) as the *significance score *to rank the candidates. In the evaluation, we always used the significance score unless explicitly stated otherwise.

## Authors' contributions

HMK and SB developed the alignment formulation and score statistics. HMK performed the SAMPI experiments and evaluation and wrote the manuscript. AW provided the mass spectrometry data and performed the Mascot experiments. All authors proof-read and approved the manuscript.
